# Diagnosing bipolar disorders: ICD-11 and beyond

**DOI:** 10.1186/s40345-019-0177-5

**Published:** 2020-01-20

**Authors:** Emanuel Severus, Michael Bauer

**Affiliations:** Department of Psychiatry and Psychotherapy, University Hospital Carl Gustav Carus, Technische Universität Dresden, Fetscherstr. 74, 01307 Dresden, Germany

A little less than 30 years after publication of ICD-10, the 11th version of the International Classification of Diseases (ICD-11) was adopted by the World Health Assembly in Genova on the 25 May 2019. ICD-11 for Mortality and Morbidity Statistics (ICD-11 MMS) is now available online (https://icd.who.int/browse11/l-m/en). For the most part, the Clinical Descriptions and Diagnostic Guidelines for ICD-11 Mental, Behavioural and Neurodevelopmental Disorders (CDDG, “blue book”) have been finished as well, publication however is still pending (Reed et al. 2019). For professionals a draft version is accessible online after registration (https://gcp.network/en/private/icd-11-guidelines/categories/bipolar-and-related-disorders). In addition the relevant innovations and changes have been published recently in a special article (Reed et al. 2019). If and when Diagnostic Criteria for Research (DCR, “green book”) for ICD-11 will be published remains to be seen.

Looking for someone who, by his work throughout the last decades, has significantly influenced the way of how we conceptualize bipolar disorders today, *International Journal of Bipolar Disorders* is very honored to have Jules Angst’ paper in the journal commenting on bipolar disorders in ICD-11 (Angst et al. 2020).

This editorial aims at further examining some of the points raised by Jules Angst and illuminating additional aspects. In principle, as outlined by him, there has been an intended harmonization of ICD-11 and DSM-5 with respect to many content-related aspects concerning the diagnosis of bipolar disorders (Reed et al. 2019). Fortunately, at the same time, ICD-11 allows more freedom for clinical judgement, where deemed appropriate. For example, there is no precise requirement regarding the exact duration for hypomanic episodes (“at least several days”), but not for depressive, manic or mixed episodes. In addition, no precise number of symptoms is required for fulfilling the criteria for affective episodes, with the exception of depressive episodes.

Comparable to DSM-5, in ICD-11 bipolar disorders are subdivided into bipolar I and bipolar II disorder, with similar diagnostic criteria. However, mixed episode continues to exist in ICD-11. To diagnose bipolar I disorder the presence of at least one past or present manic or mixed episode is mandatory. Depressive episodes may occur, but are not obligatory. In contrast, to diagnose bipolar II disorder, the presence of at least one hypomanic and one depressive episode in the course of the illness is required. A past manic episode necessitates the diagnosis of bipolar I disorder. Moreover, a single (hypo) manic (up to now: F30) or a mixed episode (up to now: F38) do not qualify for independent diagnoses any longer. The same holds true for recurrent hypomanic episodes (up to now: F31), in the absence of depressive episodes.

In line with DSM-5 cyclothymic disorder is now part of “Bipolar and Related Disorders”, with similar diagnostic criteria, but it differs from DSM-5 in so far that recurrent hypomanic symptoms may fulfill the criteria for hypomanic episodes.

In analogy to DSM-5 bipolar I or bipolar II disorder can also be diagnosed in ICD-11 in case of hypomanic, manic or mixed episodes being triggered by antidepressant treatment. However, the CDDG draft suggests that in this case one or more depressive episodes must have been present in the past. Regarding symptom criteria for a hypomanic or manic episode increased activity, in combination with euphoric, irritable or expansive mood constitutes the obligatory entrance criteria—which is supported by recent research (Merikangas et al. 2019).

In light of the above, it can also be stated that, similar to DSM-5, the diagnosis of bipolar I disorder may also be made in ICD-11, even if only the manic pole of the disease prevails at the time of diagnosis, in the form of one or more manic episodes. Although there may be good reasons content wise to proceed in this way, these are not undisputed (Angst et al. 2020)—and the definition remains confusing, not only for patients.

It also remains an open question whether the diagnosis of bipolar II disorder, although clinically helpful, is indeed an independent, valid disease entity, as postulated by ICD-11—or whether it represents, at least in part, primarily the result of methodological differences concerning the diagnosis of bipolar I and bipolar II disorder. For example, it is a matter of debate whether the course of the illness in bipolar II disorder, which is, in comparison to bipolar I disorder, predominantly characterized by depressive symptomatology, may simply be the logical consequence of the fact that the previous disease course of bipolar II disorder, compared to bipolar I disorder, as defined by the diagnostic "inclusion" criteria, also favors the depressive pole. It can also be questioned whether the difference in comorbidity with borderline personality disorder (Fornaro et al. 2016) and mood instability (Faurholt et al. 2019) between bipolar I and bipolar II disorder is not primarily due to a generally different diagnostic assessment between these two disorders (Bipolar I: on the occasion of acute treatment need for a manic or mixed episode; Bipolar II: retrospective assessment of a hypomanic episode on the occasion of an acute need for treatment for a depressive episode), possibly resulting in a higher rate of "false positives" for hypomanic episodes—and therefore bipolar II disorder. In addition, up to now, being diagnosed as bipolar I or bipolar II was, to some extent, dependent on the therapeutic approach (Severus and Bauer 2014). In this regard, ICD-11 has been helpful, as we are now able to diagnose a manic episode with a corresponding degree of severity even if the duration of symptoms is shorter than 1 week, due to treatment.

How will diagnostic criteria develop in the future, beyond ICD-11, with regard to "Bipolar and Related Disorders"? As long as diagnostic criteria continue to be primarily based on the traditional exploration of the psychopathological findings and illness history, progress towards more reliable and more valid disease entities seems to be limited. Against this background, the possibilities of ambulatory monitoring and digital phenotyping, with the option of continuous passive multimodal acquisition of objective behavioral parameters in “real time” and "real-life", are becoming increasingly important (Faurholt Jepson et al. 2018). Bipolar disorders seem to be a particularly well-suited candidate for this purpose, with their disease episode-dependent changes of motor activity and energy as major symptoms (Merikangas et al. 2019). In the long run, it could become possible to arrive at a largely rater-independent precise examination of brain function by operationalizing these as well as other essential items of the psychopathological assessment and, in combination with the RDoC initiative, to more valid mental illness entities (Torous et al. 2017). In this process, ethical issues are manifold in nature and require extensive and continuous discussion and evaluation.

## Data Availability

Not applicable
